# The impact of resilience on psychological outcomes in women after preeclampsia: an observational cohort study

**DOI:** 10.1186/1477-7525-11-194

**Published:** 2013-11-14

**Authors:** Eva Mautner, Christina Stern, Maria Deutsch, Eva Nagele, Elfriede Greimel, Uwe Lang, Mila Cervar-Zivkovic

**Affiliations:** 1 Department of Obstetrics and Gynaecology, Medical University of Graz, Augenbruggerplatz 14, Graz A-8036, Austria

**Keywords:** Preeclampsia, Depression, Post-traumatic stress, Quality of life, Psychological outcomes, Resilience

## Abstract

**Background:**

Preeclampsia is a frequent obstetric complication which affects the mother`s and the fetus’s health and can be life threatening. It also has an impact on psychological outcomes. There may be protective variables such as resilience shielding against psychosocial distress in women experiencing these pregnancy complications. The aim of this study was to examine differences in resilience in terms of quality of life, depression and post-traumatic stress symptoms in women after preeclampsia.

**Methods:**

Four international validated questionnaires were used to measure the psychological outcomes (Medical Outcome Study Short-Form SF12, Edinburgh Postnatal Depression Scale EPDS, Resilience Scale RS13, Impact of Event Scale IES-R). Statistical analyses were performed using independent-samples t-test and chi-square test.

**Results:**

67 women with previous preeclampsia returned the questionnaires. Women with high resilience showed significantly less depression (p = 0.001) and better mental quality of life (p = 0.002) compared to women with low resilience. No group differences were found on the medical and socio-demographic characteristics.

**Conclusions:**

Resilience has an important impact on the psychological outcomes in women after preeclampsia. A screening for resilience, depression and quality of life may be appropriate to identify these women.

## Background

Preeclampsia, along with other hypertensive disorders of pregnancy occurs in 2 to 8% of pregnancies. It is a major contributor to maternal mortality worldwide [1-3]. The multifactorial pathogenesis of different preeclampsia phenotypes has not been fully elucidated, therefore prevention and prediction are still not possible, and symptomatic clinical management is mainly directed to prevent maternal morbidity and mortality. Preeclampsia affects the health of the mother and the fetus seriously and may lead to maternal multiorgan dysfunction and uteroplacental insufficiency followed by intrauterine growth restriction and fetal asphyxia. Preeclampsia turns a pregnancy into a high-risk one. Women have to cope with a serious, often life-threatening illness that affects not only themselves but also their unborn [4].

Women after preeclampsia often experience more health complaints than women with uncomplicated pregnancies and seem to be impaired in their physical and mental well-being [5]. In previous studies the risk for preterm delivery was found especially to be related to low health related quality of life (HRQoL) and a high level of depressive symptoms during pregnancy and the postpartum period [6,7]. HRQoL measures the subjective psychological and physical outcomes of patients and is used to evaluate treatment regiments in medicine. It has been established as an outcome variable and health status indicator in medical and public health research [8,9]. Depressive symptoms are another negative psychological outcome of childbirth for women in different countries. A depressive condition after childbirth is characterised by sad mood, lack of interest, anxiety, sleep difficulties, reduced self-esteem, somatic symptoms such as headache and weight loss, and difficulty coping with day-to-day tasks. Adverse effects on the mother-infant relationship are well documented. Mothers with depression were less sensitively attuned to their infants and were less affirming and more negating of infants’ experiences. Depression after childbirth occurs between 10-20% in childbearing woman [10-12].

There are some studies in western and non-western countries, which focus on Post-Traumatic Stress Disorder (PTSD) as an outcome measure after childbirth with prevalence between approximately 1% and 7% at different time points after delivery [13,14]. PTSD is characterised by three symptom categories: Persistent re-experiencing of the traumatic event, persistent avoidance of stimuli associated with the event, numbing of general responsiveness, and symptoms of increased arousal [15]. According to Olde et al. [16], risk factors for PTSD in response to childbirth included a history of psychological problems, trait anxiety, the obstetric procedures, negative aspects in staff-mother contact, feelings of loss of control over the situation and lack of partner support [16]. Yehuda suggested that resilience and vulnerability factors including previous exposure to trauma, the intensity of the response to acute trauma, personal characteristics and neuroendocrine changes, such as lower cortisol levels, may be more important in the development of PTSD after a less severe traumatic event than after a severe one [17]. This might be applicable to the childbirth experience.

Resilience is defined as the ability to successfully withstand a threatening and challenging situation. It also refers to recovery from extreme distress and trauma [18]. From a conceptual perspective, resilience is associated with curiosity and intellectual skills and the ability to cope with problems [18,19]. Wagnild & Young [20] postulated a two-dimensional structure of resilience formed by two factors: "Acceptance of Self and Life" and "Personal Competence" [20]. In other investigations protective resources were classified as psychological-internal characteristics, support from family and friends, and external support systems [21]. Three domains were identified which may impact on resilience by enhancing mental health in general after exposure to adversity. These domains are secure attachment, positive emotions and a purpose of life [22]. Resilient people are more flexible than vulnerable, and they are able to protect themselves against distress by activating various protective mechanisms. It also applies to the capacity to mobilize resources [21]. To date it has not been examined whether there are differences in terms of resilience after preeclampsia.

In this study we investigated resilience as a protective personal resource regarding psychological outcomes. The aim was to examine differences in resilience in terms of post-traumatic stress symptoms, depression and HRQoL after preeclampsia. We hypothesized that resilient women have fewer PTSD symptoms, less depression and better HRQoL.

## Methods

### Sample

A total of 77 women with previous preeclampsia in pregnancy participated. Of those, 10 women were excluded from the analysis because they did not return the questionnaire or refused to take part.

The mean age of all women was 32.3 years (SD 4.86) with a range from 23 to 43 years. 16.4% of the women had experienced a previous mild preeclampsia, 71.6% severe preeclampsia and 11.9% superimposed preeclampsia. Most of the women (79.1%) had had a caesarean section: 16.4% had been declared as emergency caesareans. 40.3% of the babies after delivery had been with their mothers in the postpartum room, 50.8% had been in the intensive care unit and 6 of the babies had died. Nearly all of the women were in a relationship at the time of the survey (97.1%). The majority of women had post-compulsory school education (61.2%) or a university degree (34.3%). 38.8% of the respondents were pregnant after their pregnancy with preeclampsia at the time of the survey and 61.2% were not pregnant.

A comparison of participants with non-participants with respect to age, hypertensive disorders, gravidity, parity, time interval between index pregnancy and survey, and neonatal outcomes showed no statistically significant differences (p values were > 0.23).

### Study design

The study was conducted at the outpatient unit for hypertensive pregnancy disorders in a major university hospital in Austria. Women with a history of preeclampsia were invited to participate in this study examining adjustment after this event. Hypertensive disorders were defined according to the German guidelines [23] based on American and Australian classifications and the criteria set by the International Society for the Study of Hypertension in Pregnancy ISSHP, [24]. Inclusion criteria were German-speaking women who had been diagnosed with preeclampsia during their pregnancy and a birth during the last four years. The four year time interval was chosen for practical reasons to get a bigger sample due to the special selective clinical group we investigated. Exclusion criteria were illiteracy in German and severe mental impairment.

Eligible participants were approached from December 2009 to September 2011. After explaining the purpose of the study, participants received an informed consent form and the German questionnaire battery in the outpatient setting. No financial compensation was offered to the women for their participation.

The study protocol was approved by the local Ethics Committee of the Medical University Graz, and informed, written consent was obtained from all participants.

### Measures

Four validated questionnaires were used to measure resilience, post-traumatic stress, depression and HRQoL. All participants had to answer the questionnaires concerning their actual mental and physical quality of life, depressive symptoms and posttraumatic stress symptoms regarding their pregnancy with preeclampsia. Resilience was measured by asking women how in general they think and behave.

### Resilience Scale (RS-13)

The 13-item questionnaire measures resilience on a 7-point scale (alpha = 0.90). Women respond to the items regarding the extent to how much they agree with the different statements from 1 "I do not agree" to 7 "I agree completely". The RS-13 is the short German version of the RS-25 from Wagnild & Young, [20]. Scores range from 13 to 91 where higher scores indicating higher resilience. Based on reference groups women with less than 72 points on the resilience scale (RS-13) were defined as women with low resilience. Highly resilient women had scores above or equal to 72 [25].

The German version of the **Impact of Event Scale (IES-R)** was used to measure post-traumatic stress symptoms [26]. The 22-item questionnaire measures the severity of intrusion (7 items, alpha = 0.90), avoidance (8 items, alpha = 0.79) and hyper-arousal symptoms (7 items, alpha = 0.90). Items were rated on a 4-point scale according to how frequently each symptom had occurred during the last week from 0 'not at all’ to 5 'often’. Possible scores range from 0 to 35 on the intrusion scale, 0 to 40 on the avoidance scale, and 0 to 35 on the hyperarousal scale. A cut-off point for a PTSD diagnosis was calculated for the German version of the IES-R. A score > 0 indicates the presence of PTSD [26].

The **Edinburgh Postnatal Depression Scale (EPDS)** was administered to measure depressive symptoms. It is a 10-item screening scale developed for use in the postpartum period and validated for pregnancy [10-12]. Items inquire about mother’s mood in the past seven days and are rated on a 4-point scale ranging from 0 to 3. A cut-off point of 10 has been shown to have a sensitivity of 84% to 100% and a specificity of 76% to 88% when compared to a diagnosis of minor and major depression using a psychological interview such as the DSM-V [27]. For an Austrian population, a cut-off point of 10/11 is recommended [28].

**The Medical Outcome Study Short-Form-12** (German version) was used to assess HRQoL [29]. It measures physical and mental health by means of two summary scores: a physical component summary (PCS) and mental component summary (MCS) [30]. According to the test manual, standardized scores (M = 50, SD = 10) were calculated where higher scores indicate better health status [28].

We collected the medical and socio-demographic information of the study sample using a socio-demographic data sheet which included age, education, mode of delivery, gravidity, parity, neonatal outcomes and week of gestation.

### Statistical analyses

Data were analysed using Statistical Package for the Social Sciences (SPSS) version 16.0. Descriptive statistics were used to characterize the study sample in terms of socio-demographic and clinical variables. The independent-samples t-test was conducted to test for differences between the two resilience groups (high and low) in age. The chi-square test was used to test for between-group differences in education, hypertensive disorder groups (mild preeclampsia, severe preeclampsia, and superimposed preeclampsia), mode of delivery (planned caesarean, emergency caesarean, spontaneous vaginal), gravidity, parity, time interval from index-pregnancy to time of the survey (up to 1 year vs. more than 1 year), and neonatal outcome (postpartum room, intensive care unit, perinatal mortality). If possible, Fisher’s exact test was calculated.

An independent samples t-test was performed to explore differences in terms of HRQoL depression and the scales of post-traumatic stress disorders (intrusion, avoidance and hyper-arousal) by level of resilience (low vs. high) The level of significance was determined with p < 0.05.

## Results

The clinical and demographic characteristics of the study participants are presented in Table 1. No statistically significant group differences were found on the demographic and clinical characteristics.

**Table 1:** Demographic and clinical characteristics by level of resilience

	**Low resilience (n = 27)**	**High resilience (n = 40)**	***p***
*Age* (M ± SD)	31.7 ± 4.6	32.7 ± 5.1	0.40
*Education*			1.00
Compulsory	12 (44%)	19 (48%)	
Higher education/university	15 (56%)	21 (53%)	
*Hypertensive disorders*			0.14
Mild preeclampsia	2 (7%)	9 (23%)	
Severe preeclampsia	20 (74%)	28 (70%)	
Superimposed preeclampsia	5 (19%)	3 (8%)	
*Mode of delivery*			0.96
Planned caesarean	16 (59%)	25 (63%)	
Emergency caesarean	5 (19%)	7 (18%)	
Spontaneous vaginal	6 (22%)	8 (20%)	
*Gravidity*			0.44
Not pregnant	15 (56%)	27 (68%)	
Pregnant	12 (44%)	13 (33%)	
*Parity*			1.00
Primipara	21 (78%)	32 (80%)	
Multipara	6 (22%)	8 (20%)	
*Time interval*			0.62
Up to 1 year	9 (33%)	16 (40%)	
More than 1 year	18 (67%)	24 (60%)	
*Neonatal outcome*			0.80
Postpartum room	10 (37%)	17 (43%)	
Intensive care unit	15 (56%)	19 (48%)	
Perinatal mortality	2 (7%)	4 (10%)	

Table 2 shows group differences in the psychological outcomes measures. Patients with higher resilience showed a significant better mental health state on the SF-12 (p = 0.01) and significantly lower depressive symptoms as measured by the EPDS (p = 0.01). The mean depression score of women with less resilience (M = 11.5, SD = 6.2) was above the cut-off score of 10/11 for Austrian women, indicating more depressive symptoms for women with low resilience following preeclampsia compared to women with high resilience (M = 6.8, SD = 5.1).

**Table 2:** Differences on the SF12, EPDS, and PTSD symptoms for low and highly resilient women after preeclampsia

	**Low resilience (n = 27)**	**High resilience (n = 40)**		
	***M*** **±** ***SD***	***M*** **±** ***SD***	***p***	***Eta***^***2***^
SF-12 Physical	47.5 ± 9.2	48.1 ± 7.5	0.78	0.00
SF-12 Mental	40.8 ± 12.7	49.9 ± 10.3	<0.01	0.14
EPDS	11.5 ± 6.2	6.8 ± 5.1	<0.01	0.15
IES Intrusion	10.7 ± 7.1	11.2 ± 9.1	0.80	0.00
IES Avoidance	9.6 ± 6.7	7.3 ± 6.4	0.16	0.03
IES Hyperarousal	10.7 ± 8.6	8.3 ± 6.5	0.19	0.03

On the remaining scales, no statistically significant differences were found with respect to the resilience groups.

On the screening scale of the IES-R scale, two patients (2.9%) of the whole sample scored above the cut-off value indicating a high level of PTSD (not in the tables).

## Discussion

The development of preeclampsia was mentioned as a stressful and life threatening event in pregnancy for women and their partners [4-6]. In our study population 8.9% of the women had experienced the death of a child as a consequence of preeclampsia. Additionally, 50.8% of the babies had to be treated in the intensive care unit, so women were separated from their babies after delivery. Women had in most cases to deal with high blood pressure and recovery after caesarean section.

Prior to the diagnosis of preeclampsia, many of these women may have never experienced a significant illness and had been expecting a routine, normal pregnancy. There is evidence, that the severity of preeclampsia has a decreasing impact on HRQoL, as recent data show [31]. Women with preeclampsia are impaired in their physical and emotional well-being, such as depressive symptoms [5] and have to deal with feelings of loss of control over the situation [16].

In this study we compared differences in psychological outcomes (post-traumatic stress, depression and HRQoL) as a function of resilience in women who experienced preeclampsia. Resilience is not merely characterized by the absence of psychopathology but is the dynamic process that enables the individual to successfully adapt to severe adversity over the life course [22].

The strength of the study is the use of well known, international, validated questionnaires as screening tools for post-traumatic stress disorders, depressive symptoms, HRQoL and resilience. Furthermore this is the first study which evaluates resilience in the context of adverse pregnancy outcomes. This study may improve the understanding of resilience as a protective resource after preeclampsia and therefore may help to develop strategies to prevent negative psychological outcomes.

To cope with the severe disease in pregnancy, women need adjustment strategies because of the change from a normal into a high-risk pregnancy [4,6]. Women usually get external support during this stage of life. The support of a partner was mentioned as an especially important protective resource against the development of PTSD [16]. We only found 2.9% with PTSD symptoms above the cut of score. This is consistent with findings in the literature [13,14].

Nevertheless, we found that women with low-resilience showed significantly more depressive symptoms following preeclampsia. Moreover, women with lower resilience showed depressive symptoms above the screening cut-off score of 10/11 recommended for Austrian population [28]. It is important to note that the validation study for the German sample is very small consequently the recommended cut-off point must be used with caution. However, highly resilient women showed clearly less depressive symptoms in our population compared to low resilient women. Women with low resilience had also decreased HRQoL on the mental component scale. Based on our results we conclude that resilience may be a significant factor, which protects against emotional distress (e.g., reduce depressive symptoms and improve mental quality of life) after preeclampsia.

We are aware of a relatively small sample that limits the findings of this study. On the other hand this is a clinical group of women we examined and there are not large numbers of them. Another limitation is that the time-interval of index-pregnancy to survey is up to 4 years. However, pregnancy is a unique time in the life of any woman [32], and consequently, recall of pregnancy-related events in women even 30 years or later after delivery has been shown to be reproducible and reasonably accurate [31,32]. In this study we focused on the outcome parameters HRQoL, depressive symptoms and PTSD-symptoms. In further studies other parameters e.g. anxiety, coping strategies or special interventions could be investigated to get a better insight into emotional distress after preeclampsia.

### Clinical implications

It is important to examine coping strategies after preeclampsia and being able to offer adequate supportive interventions when they are needed [16].

Women seek information and explanations after this challenging event. Receiving relevant information about their medical and psychological condition gives women an active role and the possibility to cope with their illness and get control over the situation [14,21]. Woman will benefit from educational interventions on common responses and adaptive reactions in the acute situation and in the subsequent years after this burdened pregnancy. Consequently, the personal competence of women who have had preeclampsia can be improved, and is therefore an important factor in improving resilience [20].

Resilience is changeable and can increase during psychological treatment and leads to more well-being [22,25]. Enhancing the ability to experience positive emotions could play an important role in making people more resilient to depression. A meta-analysis established that interventions as diverse as writing gratitude letters, practising optimistic thinking, replaying positive experiences and socializing have beneficial effects on levels of depression [22]. Also, mediation-based or mindfulness-based approaches may be promising venues in increasing positive emotions. Additional purpose of life was found to buffer against the negative effects of life-threatening physical illness on mental health in general. A sense of meaning and a purpose in life is something very personal. Prolonged meditation or mindfulness training, in which people are trained to continuously focus their attention on the present moment, may result in an increased awareness of meaning and purpose experienced in daily life situations [22]. Furthermore, supportive responses from others help individuals to overcome adversity and increase growth following adversity. For these reasons, a special multi-professional outpatient department offering medical and psychological support might improve the HRQoL and well-being after preeclampsia. Especially women with low-resilience will benefit from support after this event and in further pregnancies.

## Conclusions

Resilience is a protective variable after preeclampsia indicating low levels of depression and better mental quality of life. The screening of resilience, depression and HRQoL may be appropriate to identify women who will benefit from psychological support after this event and in further pregnancies. Resilience will improve with interventions that increase the personal competence of women and enable them to gain self-confidence and control over their disease.

## Competing interests

The authors declare that they have no competing interests.

## Authors’ contributions

All authors made substantive contributions to the present study. EM, CS, MD, MC developed the original concept of the study, recruited patients and collected data. EM and CS analyzed the Data. EM, CS, EN, EG, UL drafted the manuscript. EM revised the manuscript. All authors have given final approval to the submitted version.
